# Understanding the Design and Sensory Behaviour of Graphene-Impregnated Textile-Based Piezoresistive Pressure Sensors

**DOI:** 10.3390/s25072000

**Published:** 2025-03-22

**Authors:** Md Faisal Mahmud, Md Raju Ahmed, Prasad Potluri, Anura Fernando

**Affiliations:** Department of Materials, The University of Manchester, Oxford Rd, Manchester M13 9PL, UK; mdfaisal.mahmud@postgrad.manchester.ac.uk (M.F.M.); prasad.potluri@manchester.ac.uk (P.P.)

**Keywords:** graphene, nonwoven textiles, piezoresistive pressure sensors, flexible electronics, silver-coated electrodes, wearable sensors

## Abstract

Graphene-based textile pressure sensors are emerging as promising candidates for wearable sensing applications due to their high sensitivity, mechanical flexibility, and low energy consumption. This study investigates the design, fabrication, and electromechanical behaviour of graphene-coated nonwoven textile-based piezoresistive pressure sensors, focusing on the impact of different electrode materials and fabrication techniques. Three distinct sensor fabrication methods—drop casting, electrospinning, and electro-spraying—were employed to impregnate graphene onto nonwoven textile substrates, with silver-coated textile electrodes integrated to enhance conductivity. The fabricated sensors were characterised for their morphology (SEM), chemical composition (FTIR), and electromechanical response under cyclic compressive loading. The results indicate that the drop-cast sensors exhibited the lowest initial resistance (~0.15 kΩ) and highest sensitivity (10.5 kPa^−1^) due to their higher graphene content and superior electrical connectivity. Electro-spun and electro-sprayed sensors demonstrated increased porosity and greater resistance fluctuations, highlighting the role of fabrication methods in sensor performance. Additionally, the silver-coated knitted electrodes provided the most stable electrical response, while spun-bonded and powder-bonded nonwoven electrodes exhibited higher hysteresis and resistance drift. These findings offer valuable insights into the optimisation of graphene-based textile pressure sensors for wearable health monitoring and smart textile applications, paving the way for scalable, low-power sensing solutions.

## 1. Introduction

The rapid progress in flexible and wearable pressure sensors has been largely driven by the incorporation of electronic textiles (e-textiles) into areas like health monitoring, robotics, and smart wearable devices [1]. Among these technologies, piezoresistive sensors that utilise graphene have gained attention due to their remarkable sensitivity to mechanical forces, quick response times, and low energy requirements, making them ideal for wearable applications. Graphene’s distinctive properties, such as high electrical conductivity, flexibility, and stability, further enhance the performance of these sensors [2]. In piezoresistive pressure sensors, variations in current or resistance occur in response to compression, enabling the detection of mechanical deformation or external stimuli [3,4]. One of the key challenges in advancing pressure sensors is finding a balance between stability and sensitivity, which is heavily influenced by the choice of conductive materials and the substrate [5,6]. To tackle this issue, researchers have explored materials like low-dimensional carbon-based options (e.g., carbon black, carbon nanotubes, and graphene), metallic nanomaterials (e.g., silver nanowires, silver nanoparticles, and gold nanowires), and conductive polymers (e.g., PANI, PEDOT: PSS, and MXene) [7]. Among these, silver nanowires and graphene stand out due to their exceptional piezoresistive properties, excellent electrical performance, and mechanical flexibility, making them particularly suitable for strain sensor applications [8].

Graphene, especially when used as a conductive ink, shows great potential for sensing applications because of its unique electron transport capabilities, allowing it to rapidly detect resistance or current changes. For instance, Ren and colleagues demonstrated that cotton fabric coated with reduced graphene oxide (rGO) maintained impressive bending stability under compressive strain, showcasing its potential for use in flexible textile sensors [9]. These developments highlight the growing promise of graphene-based materials in creating the next generation of wearable pressure sensors. Nonwoven electro-textile materials, recognised for their fine fibres and intricate mesh structures, have emerged as exceptional piezoresistive sensors, offering the ability to detect minute stresses, strains, and vibrations with remarkable sensitivity [9]. The performance of nonwoven-based graphene-coated piezoresistive pressure sensors was greatly enhanced due to the well-structured porous arrangement, improving sensitivity and an expanded working range [10].

Recent progress in this field has illuminated their potential for a broad spectrum of applications. For example, Jain and Chatterjee successfully created reduced graphene oxide (rGO)-coated nylon nonwoven fabrics through optimised dip-coating techniques. This innovation achieved impressive electrical conductivity (3.62 kΩ/sq.), durability, and piezoresistive properties, making these fabrics ideal for low-pressure tactile sensing uses [11]. Similarly, Wang et al. (2018) demonstrated the effectiveness of nano-silver-infused nonwoven fabrics in wearable piezoresistive sensors, achieving high sensitivity, low noise levels, and dependable performance for long-term monitoring of cardiorespiratory signals [12]. Other notable advancements include the work of Lu et al. (2019), who integrated rGO and PDMS with polyester nonwoven substrates to develop a 3D piezoresistive pressure sensor. This sensor featured high sensitivity (35.37 kPa^−1^), durability over 1200 cycles, and the ability to track various physiological activities, such as breathing and joint movements [13]. Researchers have also delved into advanced nanomaterials and scalable manufacturing processes. Yu et al. (2022) leveraged Ti3C2Tx MXene-coated nonwoven fabric composites to achieve a sensor with high sensitivity (6.31 kPa^−1^), a broad sensing range (150 kPa), and durability exceeding 2000 cycles, positioning it as a promising candidate for wearable medical diagnostics and electronic skin applications [14]. Zhang et al. (2021) contributed by developing silver nanowire-coated cotton nonwoven fabrics for flexible pressure sensors. Their innovations delivered high sensitivity (7.41 kPa^−1^), fast response times (<50 ms), and enhanced durability through in situ conductive network synthesis, underscoring the potential of these sensors in wearable electronics and environmentally sustainable technologies [15].

Ahmed et al. (2023) advanced durability further by producing machine-washable graphene-coated nonwoven fabrics using a novel isophorone-based quaternary compound. This method achieved exceptional electrical conductivity (140 Ω/sq) and high sensitivity (44.59 kPa^−1^) over multiple washing cycles [16]. Meanwhile, Hasan et al. (2019) enhanced the hydrophilicity of nonwoven polypropylene fabrics via oxygen plasma treatment, facilitating uniform graphene oxide coating. Their approach achieved a sensitivity of 0.050 kPa^−1^ across a wide pressure range (0–60 kPa) and demonstrated scalability for industrial production [17]. More recently, Zhang et al. (2024) introduced graphene sterically wrapped textile-based piezoresistive sensors through a spray-coating process. These sensors exhibited ultrahigh sensitivity (259.6 kPa^−1^), a wide sensing range (up to 1 MPa), and remarkable durability over 12,000 cycles, making them well suited for wearable electronics and ergonomic applications [18].

Despite these studies, significant gaps remain in understanding the sensory behaviour and contribution of electrodes in the sensing performance of the graphene-based piezoresistive pressure sensor and how electrode and sensing layer preparation methods influence sensor performance. All the research in this area highlights the performance and the consideration of the electrodes and their contribution. Continued research is necessary to refine fabrication techniques and gain insight into the improvement of the sensory performance of graphene-coated piezoresistive sensors on textile substrates. This research aims to understand the sensory behaviour of graphene-coated piezoresistive sensors on textile substrates and to evaluate the influence of different fabrication techniques and electrode materials. However, while existing studies have examined various nanomaterials and nonwoven structures, gaps remain in understanding the impact of electrode materials and graphene layer preparation methods on sensor performance.

## 2. Experimental Section

### 2.1. Materials

Graphene nanoparticles used in this present study were provided by First Graphene Ltd., (Manchester, UK) PureGRAPH 10 flakes. It has a tapped density of 0.124 g/cm^3^. Polycaprolactone ACS reagent ≥ 99.9% and polyurethane diol solution obtained from the Textiles Composites Analysis Laboratory, University of Manchester, were provided by Sigma-Aldrich, Dorset, UK, and have a density of 1.18 g/ML. A nanoporous electro-spun nonwoven was prepared using a polyurethane diol solution. Spun-bonded nonwoven fabrics, with a thickness of 0.61 mm and an areal density of 60 g/m^2^, were produced from a 1:1 polypropylene/polyester fibre blend sourced from Landor. Electrodes for sensor fabrication were procured from HITEK LTD. (Scunthorpe, UK). The specifications of the silver-coated textile-based electrodes have been shown in Table 1.

### 2.2. Preparation of Graphene Ink

**Figure 1:**
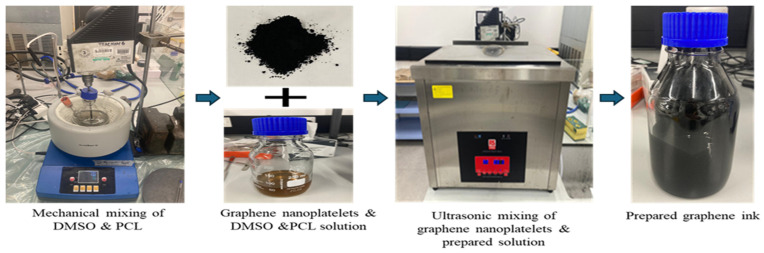
Graphene ink preparation process.

Two procedures were followed to prepare graphene ink: To begin, for 48 h, a mixture of 500 mL of dimethyl sulfoxide and 50 g of polycaprolactone was stirred at room temperature using a mechanical stirrer set at 70 °C. A mixture of 7 g of graphene flakes and 293 g of solvent was made and mixed thoroughly. Effective preparation of the graphene ink was performed through the sonication process for nine hours. Graphene ink preparation process is shown in Figure 1**.**

### 2.3. Preparation of Graphene-Impregnated Piezoresistive Sensing Layer

**Figure 2:**
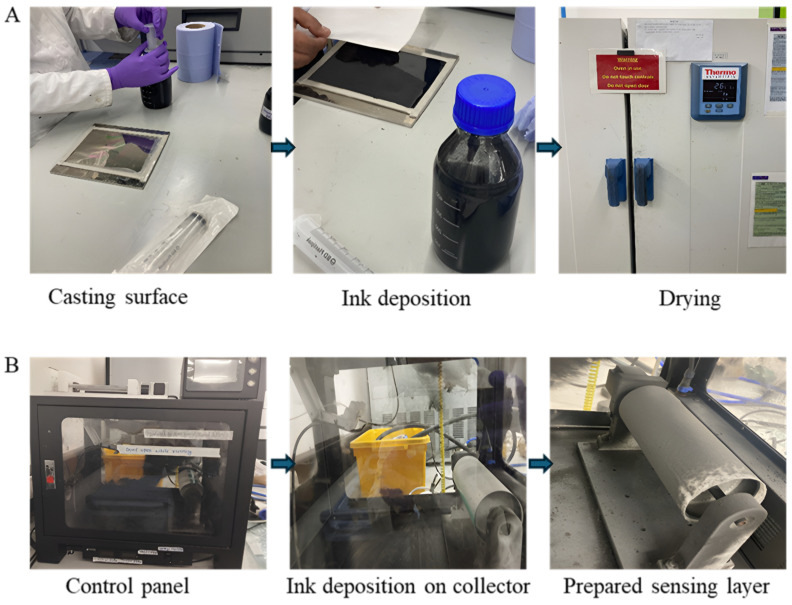
Graphene-impregnated piezoresistive sensing layer preparation: (**A**) the drop-casting method and (**B**) electrospinning and electro-spraying.

This research prepares graphene-coated piezoresistive sensing layers via drop casting, electrospinning, and electro-spraying (Figure 2). During electrospinning, particles are moved to a nanofibre mat, and graphene ink is deposited evenly with the right contact concentration and viscosity. Drop casting creates a thin graphene sensing layer for the piezoresistive pressure sensor. Graphene particles are electro-spun and electro-sprayed using the JDF05 electrospinning machine, Guangxi, China. Detailed parameters of the electrospinning are shown in Table 2. The parameters were adjusted to avoid ballooning effects and maintain the graphene droplets’ uniform size. The applied voltage results in higher electrostatic force to stretch the jet, preventing agglomeration of graphene during electrospinning. The polyurethane diol solution is nanoporous out of a syringe at high voltage. Solid polymer fibres containing graphene form when the solvent evaporates. A coherent fibrous mat from randomly interwoven fibres forms on the collector. Electro-spraying achieves accurate graphene nanoparticle coating forms and distribution. This procedure uses the same graphene ink as electrospinning. The ground collector applies graphene ink to both sides of spun-bonded polyester nonwoven fabric. Graphene particles link to polyester to form a thin, permeable sensor layer.

### 2.4. Characterisation

In this research, graphene ink was chemically characterised by Fourier Transform Infrared (FTIR) spectroscopy, and the surface texture of all piezoresistive sensing layers was morphologically investigated by Scanning Electron Microscopy (SEM). The chemical composition, functional groups, and bonding structures of graphene ink were analysed using a platinum ATR-FTIR machine. The scan was set to span a standard wavenumber range from 4000 cm^−1^ to 400 cm^−1^, focusing on relevant functional groups. Transmittance spectrums were generated to identify chemical bonds and functional groups. Surface morphologies of drop-cast, electro-spun, and electro-spraying-based piezoresistive sensing layers were analysed using an SEM machine, Oregon, USA. Comparisons were made to understand graphene distribution on the nonwoven surface.

### 2.5. Fabrication of the Piezoresistive Sensing Layers with Electrodes

**Figure 3:**
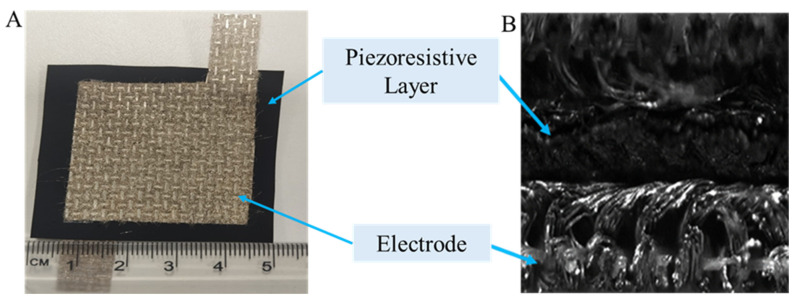
Fabrication of the piezoresistive sensing layer with the electrode: (**A**) a photograph of the fabricated sensor with the electrode and (**B**) the cross-sectional view of the sensor captured on DIC camera.

**Figure 4:**
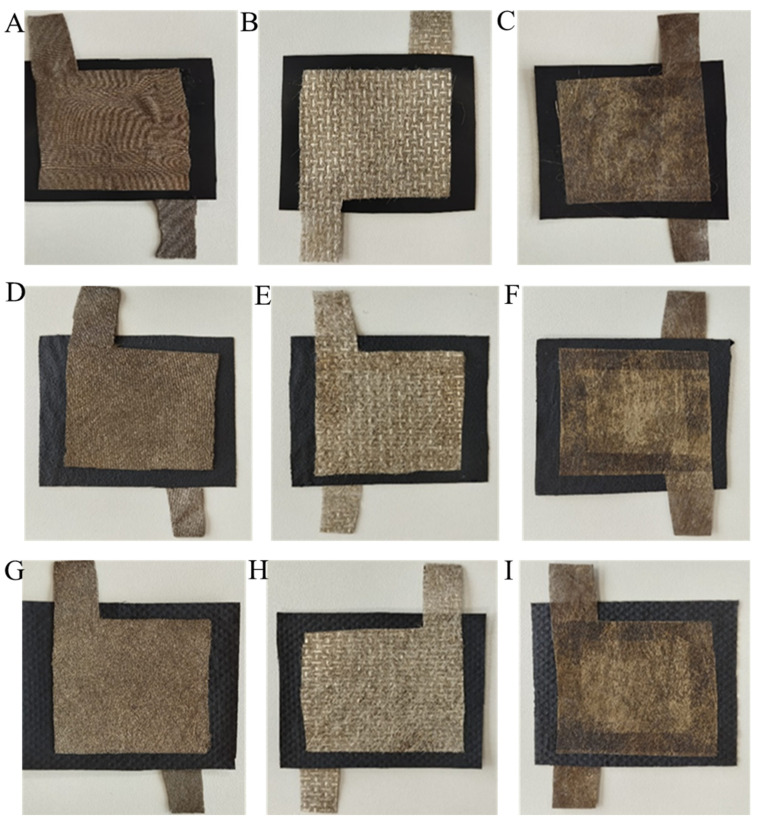
Fabricated piezoresistive sensors. (**A**) The drop-casted sensing layer with the silver-coated knitted electrode. (**B**) The drop-casted sensing layer with silver-coated powder-bonded nonwoven electrode. (**C**) The drop-casted sensing layer with the spun-bonded silver-coated nonwoven electrode. (**D**) The electro-spun sensing layer with the silver-coated knitted electrode. (**E**) The electro-spun sensing layer with the silver-coated powder-bonded nonwoven electrode. (**F**) The electro-spun sensing layer with the spun-bonded silver-coated nonwoven electrode. (**G**) The electro-sprayed sensing layer with the silver-coated knitted electrode. (**H**) The electro-sprayed sensing layer with the silver-coated powder-bonded nonwoven electrode. (**I**) The electro-sprayed sensing layer with the spun-bonded silver-coated nonwoven electrode.

The sensing layers were fabricated by diverse techniques and coupled with electrodes to evaluate circuit resistance. The material, composed of electrodes coated in silver and nonwoven textiles amalgamated with powder and spun, exhibited little electrical resistance (under 100 Ohms/m^2^), indicating its conductivity during evaluations. The sensor’s construction involved placing a piezoresistive nonwoven fabric (5 cm × 5 cm) between two silver-coated electrode layers (4 cm × 4 cm). Following the precise alignment of all layers, a garment iron was utilised to impart heat on both sides of the sensor by maintaining 60 °C temperature. A 5 mm double-sided tape is employed to adhere all layers prior to heat pressing. Figure 3 illustrates the constructed sensor together with its cross-sectional view. The details of the piezoresistive pressure sensor are shown in Table 3, and also all the fabricated piezoresistive sensors are shown in Figure 4.

### 2.6. Measurement of the Resistance of the Sensors

This research concentrated on observing the trend of electrical resistance under load to examine the piezo resistivity of sensing materials. The initial electrical resistance of each sensor was assessed with a multimeter, followed by measurements during the dynamic compression loading process utilising a half-bridge circuit. The alteration in the electrical resistance of the graphene-based piezoresistive sensing layers was recorded using LabVIEW (Version: 2023,Q3) software (NI-9219).

#### 2.6.1. Initial Resistance Measurement of the Sensors

By using a digital multimeter, Everett, DC, USA, the initial resistance values of all fabricated sensors were measured. After turning it on, the multimeter was set to resistance mode. The red and black probes of the multimeter were properly connected alternatively with the electrode fabric of the sensors, and the multimeter reading was taken carefully without applying any external force on it. Five resistance readings were recorded from the multimeter by placing the probe between every 1 cm place of the sensor were taken for each sensor, and the average value was calculated for comparison.

#### 2.6.2. Resistance Measurement by Half-Bridge Circuit

**Figure 5:**
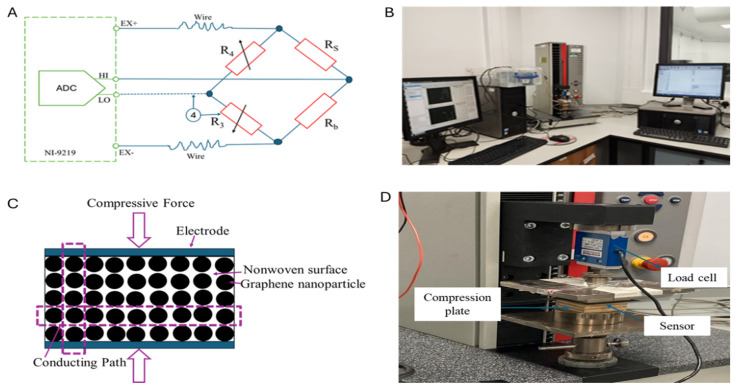
The electromechanical characterisation of the sensors, (**A**) Wheatstone half-bridge circuit diagram for resistance measurement through ADC card, (**B**) Electromechanical testing setup, (**C**) Schematic of the conducting path upon load application, (**D**) Compression application on the sensor.

Electrical resistance was measured by a half-bridge circuit in dynamic loading conditions. A resistance box and the prepared piezoresistive sensor were used to form the circuit. The electromechanical testing setup and necessary illustrations are shown in Figure 5. A data acquisition card (NI-9219) $\frac{U_{HI-LO}}{U_{Ex}}$ formula was used to measure and record the electrical resistance reading. At the beginning of the measurement, the bridge was balanced using the resistance box before starting the tests, and the balancing value (resistance) was noted for further use. With the help of the NI-9219 data acquisition card $\frac{U_{HI-LO}}{U_{Ex}}$ reading was taken, and for the calculation of the dynamic electrical resistance, the following equation was used.

$$R_{s}=R_{b}\times \frac{1-2x\frac{U_{HI-LO}}{U_{Ex}}}{1+2x\frac{U_{HI-LO}}{U_{Ex}}}$$

where *R_S_* = piezoresistive sensor resistance; *R_b_* = resistance of the box.

### 2.7. Electromechanical Quasi-Static Compression Test

Each sensor was exposed to a consistent humidity level of 65% RH ± 5% RH and a temperature of 23 ± 0.6 °C for at least 24 h prior to testing, in accordance with the specifications set out in ASTM D1776. The compression tests were performed with a Zwick/050 tensile tester in a quasi-static mode. Compression was applied between two plates measuring 4 cm by 4 cm. A 100-cycle load was implemented under cyclic loading conditions, maintaining a standard force ranging from 1 N to 80 N at a normal strain of 0.5 mm. The cycle’s velocity during the test is 3 mm/min. Resistance measurements were subsequently taken following the application of compressive force during the duration. The resistance was measured via a Wheatstone bridge configuration on a data acquisition device identified as NI-9219. Owing to time limitations, one sample from each graphene-based piezoresistive nonwoven sensor was evaluated.

## 3. Results and Discussion

### 3.1. Fourier Transform Infrared (FTIR) Spectroscopy Analysis

**Figure 6:**
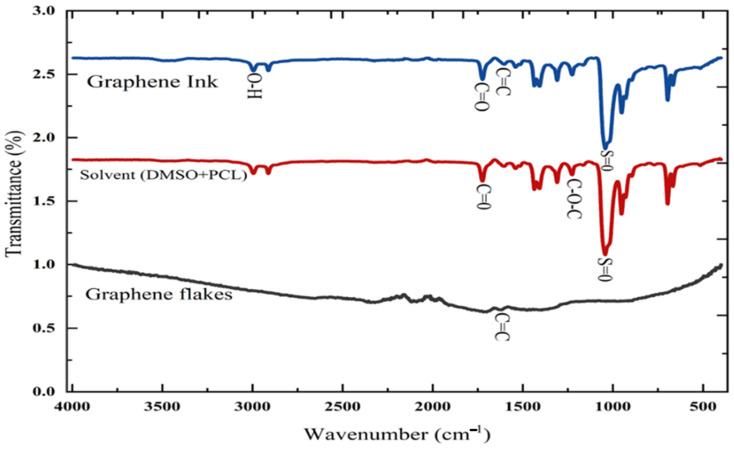
FTIR spectroscopy of graphene flakes, solvent and graphene ink.

The FTIR spectra presented in Figure 6 illustrate the characteristics of graphene and its associated functional groups. The figure presents the FTIR spectra for both graphene flakes and the solution, enabling a comparative analysis. A distinctive peak in the range of 1500–1600 cm^−1^ was observed in the graphene flakes. This peak indicates the C=C stretching in the aromatic ring. The C=C bonds in this system can be analysed in relation to the vibrational modes of carbon atoms in the sp^2^ hybridised configuration. A peak is observed in the solvent spectrum within the range of 1040–1070 cm^−1^. This peak indicates the presence of dimethyl sulfoxide (DMSO) in the solution. A subsequent peak has been observed in the range of 1720–1730 cm^−1^. This apex demonstrates the hydrogen bonding interactions between DMSO and the carbonyl groups of PCL. The peak associated with (C-0-C) in the 1170–1250 cm^−1^ range is anticipated to appear as per standard observations, indicating the presence of ester linkages in PCL. The observed change in the characteristic peaks, particularly the carbonyl stretches of PCL, indicates a significant interaction between DMSO and PCL in the solution. In the spectral range of 2850–3000 cm^−1^, peaks associated with C-H stretching vibrations from both DMSO and PCL are observed. The peak observed at 1050–1070 cm^−1^ indicates the continued presence of DMSO in the graphene ink, emphasising its interaction with the graphene flakes. The FTIR spectra of the graphene ink will exhibit distinct peaks indicative of graphene, DMSO, and PCL. This study identifies potential connections or interactions between graphene and the polymer matrix.

### 3.2. Morphological Investigation (SEM)

**Figure 7:**
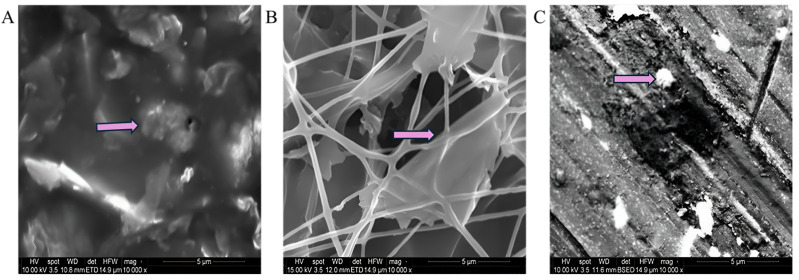
SEM image of the graphene-coated piezoresistive sensing layer of nonwoven fabric: (**A**) the drop-casted graphene sensing layer, (**B**) the electro-spun graphene-coated piezoresistive sensing layer, and (**C**) the electro-sprayed graphene-based piezoresistive sensing layer.

Scanning Electron Microscopy (SEM) analysis was performed to assess the surface texture and structural porosity of the sensing layer. The distribution of graphene across the surface was uniformly observed throughout the sensing layers. This SEM analysis enables the visualisation of the structure, distribution, and interaction of graphene on the surface of the nonwoven fabric. Figure 7 displays SEM images of the sensing layer prepared through drop-casting, electrospinning, and electro-spraying methods. SEM images were obtained at a 5 µm view field by maintaining a voltage of 10 kV and a magnification of 10,000×, the arrow marks indicate the graphene particles on the surface of the piezoresistive sensing layers. The SEM image of the drop-casting samples indicates that graphene particles are in proximity, demonstrating a high density. The SEM image confirmed that the drop-casting sample displays the highest amount of graphene particles. The electrospinning samples demonstrated a nanofibrous structure throughout the network. The deposition of graphene particles resulted in a distribution that was comparatively lower than that observed in the drop-casting sample. In the electro-sprayed sample, the same graphene ink was applied to the polyester nonwoven fabric using the same methods as those employed for the drop-casting and electrospinning samples. SEM image of the electro-spraying sample demonstrates the conditions of graphene deposition, showing that graphene particles are deposited on the surface of the polyester nonwoven. The visualisation of the amount of graphene in the SEM images also validates the graphene content variation in the prepared sensing layer from different techniques as shown in Table 3 and the SEM image indicates that the porosity of the samples is ordered as follows: drop casting exhibits lower porosity than electrospinning, which is less porous than electro-spraying.

### 3.3. The Initial Resistance of the Piezoresistive Sensors

**Figure 8:**
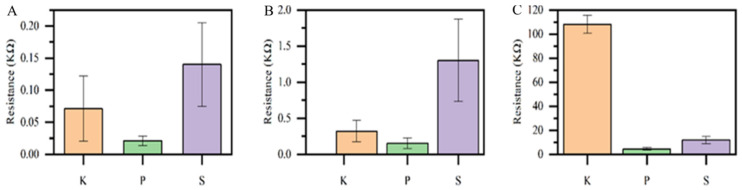
The initial resistance of the sensors prepared from: (**A**) the drop-casted sensing layer with three different electrodes, (**B**) the electro-spun sensing layer with three different electrodes, and (**C**) the electro-sprayed sensing layer with three different electrodes.

The sensors’ initial resistance was measured post-fabrication using various electrode types, as illustrated in Figure 8. The letters K, P, and S in the group column bar represent the electrode types of the sensors. “K” represents sensors made with silver-coated knitted electrodes, “P” signifies sensors constructed with powder-bonded silver-coated nonwoven fabric electrodes, and “S” indicates sensors created with spun-bonded silver-coated nonwoven fabric electrodes.

The evaluation examines the initial resistance of sensors made from different electrode types: silver-coated knitted (K), powder-bonded silver-coated nonwoven fabric (P), and spun-bonded silver-coated nonwoven fabric (S). Results show that sensors with a drop-cast sensing layer exhibit the lowest initial resistance, recorded at under 0.15 KΩ. The improved conductivity is attributed to the closer proximity of graphene particles. Conversely, sensors utilising an electro-spun sensing layer demonstrate a higher initial resistance of under 1.5 KΩ. This phenomenon is due to the reduced density of graphene particle packing within a nanoporous structure, leading to diminished conductivity. The electro-spraying samples demonstrate the highest initial resistance, exceeding 5 KΩ. This application method results in greater distances between graphene particles and improved surface porosity, subsequently decreasing conductivity. The results indicate that the method of sensor fabrication significantly affects the arrangement and density of graphene particles, which in turn influences the conductivity and initial resistance of the sensors. The drop-casting method shows greater effectiveness in achieving lower resistance values while electro-spraying leads to higher resistance due to the arrangement of graphene particles.

The ANOVA analysis shown in Table 4 was performed to assess the initial resistance values among different sensors. ANOVA evaluates variance across different test results, producing key metrics such as the ‘sum of squares’, degrees of freedom (denoted as ‘df’), ‘F’ statistic, and ‘P’ statistic. The ANOVA results show that the *p*-value indicates the significance level, while the F-value represents the ratio of explained variance to total variance. *p*-values below 0.05 for all sources demonstrate statistical significance. From Table 4, the initial resistance values yield notably small *p*-values and significantly high f-values. This demonstrates a significant influence of structural porousness and the state of graphene deposition on the conductivity and resistance of the sensors.

### 3.4. The Electromechanical Characterisation of Different Sensors

In the electromechanical characterisation of every sensor, the cyclic compressive force and corresponding resistance signals were captured to investigate the role of electrodes and the sensor quality produced using three different preparation techniques. Every sensor’s cyclic load was applied for 100 cycles over a period. In the result and discussion, changes in resistance will be interpreted over a half-cycle period and changes in the resistance of the sensors over a five-cycle period.

#### 3.4.1. The Electrical Resistance of the Prepared Sensors in Dynamic Conditions

**Figure 9:**
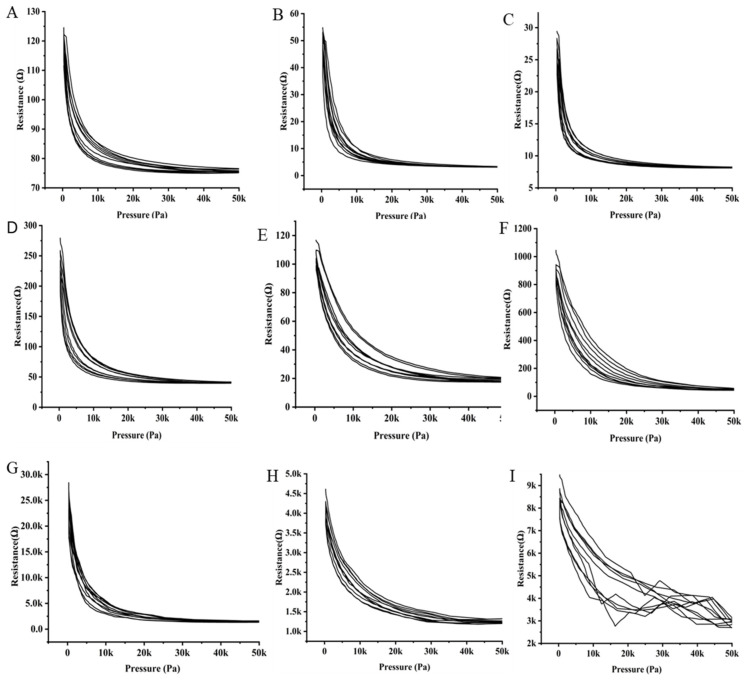
Pressure vs. resistance of the graphene-impregnated piezoresistive sensing layer over five cycles: (**A**) the drop-casted sensing layer with the silver-coated knitted electrode, (**B**) the drop-casted sensing layer with the powder-bonded silver-coated nonwoven electrode, (**C**) the drop-casted sensing layer with the spun-bonded silver-coated nonwoven electrode, (**D**) the electro-spun sensing layer with the silver-coated knitted electrode, (**E**) the electro-spun sensing layer with the powder-bonded silver-coated nonwoven electrode, (**F**) the electro-spun sensing layer with the spun-bonded silver-coated nonwoven electrode, (**G**) the electro-sprayed sensing layer with the silver-coated knitted electrode, (**H**) the electro-sprayed sensing layer with the powder-bonded silver-coated nonwoven electrode, and (**I**) the electro-sprayed sensing layer with the spun-bonded silver-coated nonwoven electrode.

Figure 9 illustrates the resistance vs. pressure characteristic curves of graphene-impregnated piezoresistive pressure sensing layers prepared from drop-cast, electro-spun, and electro-sprayed methods. All the sensing layers were fabricated using three silver-coated electrodes over five cycles. The resistance vs. pressure characteristic curves of the piezoresistive pressure sensors derive from the electrical connections made by the electroconductive graphene particles during the sensor compression. In the prepared piezoresistive pressure sensing layers, variations in current or resistance occur in response to compression, enabling the detection of mechanical deformation in the sensing layers. At the time of compression and relaxation, graphene particles in the piezoresistive sensing layer come closer and allow contact points to facilitate the reduction in the resistance followed by conductivity increment and vice versa. It can be seen in Figure 9 that all sensors show satisfactory repeatability over five cycles of continuous loading and relaxing and induce hysteresis in the resistance–pressure curves. From the induced hysteresis, it is noticeable that as pressure increased gradually, the resistance of the sensors decreased as graphene particles facilitated the conductivity of the sensors. The drop-cast piezoresistive sensing layer with the same specification showed variation in their pressure vs. resistance curve in terms of hysteresis. The silver-coated knitted electrode exhibits the least hysteresis, while the silver-coated powder-bonded and spun-bonded nonwoven electrodes display distinct hysteresis characteristics, highlighting material-dependent differences in sensor response. In the case of the electro-spun sensing layer (0.094 g), the silver-coated knitted electrode offers enhanced sensing performance, stable signal quality, and reliable electrical connectivity compared to its nonwoven counterparts as the knitted electrode enables rapid signal generation due to its structural responsiveness, which facilitates resistance signal formation upon material elongation [19]. In the electro-sprayed sensing layer, the hysteresis behaviour in the silver-coated spun-bonded electrode fabricated sensor is a more irregular pattern compared to the other two electrodes due to the least amount of graphene (0.022 g). The silver-coated knitted electrode demonstrates the lowest hysteresis, whereas the silver-coated powder-bonded and spun-bonded nonwoven electrodes exhibit distinct hysteresis characteristics, emphasising the role of electrode composition in sensor response. This can be attributed to the knitted electrode’s structural adaptability, which enhances signal generation efficiency by facilitating rapid resistance changes in response to material elongation [19] as well as the silver-coated knitted electrode can ensure a better connection between the conductive particles and the electrical circuit due to the fact it has better sensing performance compared to other silver-coated nonwoven electrodes [12]. On the other hand, powder-bonded and spun-bonded electrodes have random fibre orientation leading to inconsistent resistive response results from irregular contact between silver and graphene particles during loading and unloading conditions [20]. Among all the sensors, the drop-casted graphene-impregnated piezoresistive pressure sensor demonstrated the best resistance recovery under loading conditions due to higher graphene content (0.146 g). At the compression state, graphene contact reaches the percolation threshold, and more conductive pathways are generated from the direct contact between graphene sheets compared to the other two piezoresistive sensing layers. The drop-casted piezoresistive sensing layer with the knitted electrode showed less hysteresis in its loading and unloading conditions. As the thickness of the sensing layer is the same for all the sensors, the contribution of the electrode is noticeable, and the graphene particle density in the 50 mm× 50 mm dimension in the drop-casted sensing layer is higher than that of the other piezoresistive sensing layer. The superior performance of the drop-cast sensing layer is attributed to its higher graphene content (0.146 g), lower initial resistance, and enhanced conductivity. Additionally, the silver-coated knitted electrode ensures stable electrical connectivity, making the drop-cast graphene-coated piezoresistive sensor the most effective sensing performance [12].

#### 3.4.2. Changes in Sheet Resistance Due to Applied Pressure (0 to 50 KPa) on Graphene-Impregnated Piezoresistive Pressure Sensors over the Half-Cycle Period

The resistance changes due to applied pressure in the graphene-impregnated piezoresistive pressure sensors are shown in

**Figure 10:**
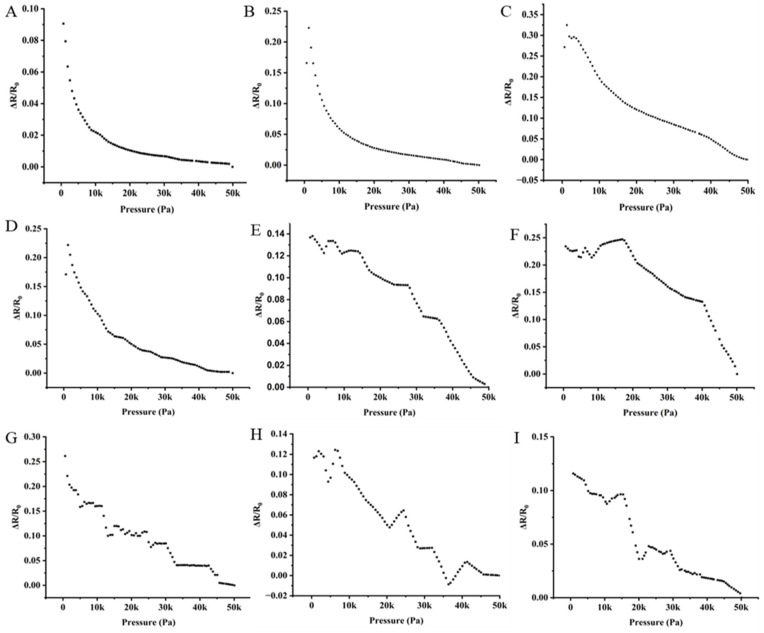
Changes in the sheet resistance due to applied pressure (0 to 50 KPa) of graphene-impregnated piezoresistive sensors over the half-cycle period: (**A**) the drop-casted sensing layer with the silver-coated knitted electrode, (**B**) the drop-casted sensing layer with the powder-bonded silver-coated nonwoven electrode, (**C**) the drop-casted sensing layer with the spun-bonded silver-coated nonwoven electrode, (**D**) the electro-spun sensing layer with the silver-coated knitted electrode, (**E**) the electro-spun sensing layer with the powder-bonded silver-coated nonwoven electrode, (**F**) the electro-spun sensing layer with the spun-bonded silver-coated nonwoven electrode, (**G**) the electro-sprayed sensing layer with the silver-coated knitted electrode, (**H**) the electro-sprayed sensing layer with the powder-bonded silver-coated nonwoven electrode, and (**I**) the electro-sprayed sensing layer with the spun-bonded silver-coated nonwoven electrode.

Figure 10 shows that as the pressure increases, the resistance decreases in the piezoresistive sensing layer due to the creation of an electrically conductive path from graphene nanoparticles. In the drop-casted sensing layers, knitted electrodes show a sharp change in resistance, while powder-bonded silver-coated nonwoven electrodes show similar resistance changes. The silver-coated knitted electrodes ensure better electrical contact with the drop-cast graphene layer, generating better-quality electrical signals from the electro-spun piezoresistive pressure sensor. The silver-coated knitted electrode (Figure 10D) demonstrated minimal resistance fluctuations, indicating stable performance due to the higher graphene (0.146 g) content in the sensing layer and the flexible structure of the knitted electrode [12], contributing to holding the superiority.

In contrast, the powder-bonded silver-coated nonwoven electrodes exhibited slight (Figure 10E) and significant (Figure 10F) fluctuations. The superior signal stability of the silver-coated knitted electrode [19] is attributed to its efficient contact with the sensing layer. Additionally, the nanoporous structure of the electro-spun layer plays a crucial role in maintaining strong connectivity between graphene particles during deformation, ultimately enhancing electrical performance.

Among the electrodes, the silver-coated knitted electrode exhibited the most stable electrical signals with minimal fluctuations for their structural flexibility (Figure 10G), followed by the powder-bonded silver-coated nonwoven electrode with slight (Figure 10I) and fewer fluctuations for random fibre orientation within the nonwoven structure create irregular contact between silver and graphene particles (Figure 10H). The superior performance of the silver-coated knitted electrode is attributed to its enhanced electrical contact between graphene and silver particles on the nanoporous electro-spun sensing layer, ensuring better signal quality.

It was observed that silver-coated knitted electrodes with drop-cast graphene-based piezoresistive sensing layers perform superiorly in terms of change in resistance and signal quality for graphene content (0.146 g) and less distance between two neighbouring graphene, ensuring quick particle contact both between graphene and with the silver particles in the electrodes. A knitted electrode can provide a fast connection to produce a signal, as the knitted structure strongly depends on generating a resistance signal upon the elongation of the materials [19]. Graphene nanoparticles on the textile surface can generate the highest resistance signal on compression from both sides of the pressure sensors.

#### 3.4.3. Analysis of the Sensitivity of Graphene-Impregnated Piezoresistive Pressure Sensors over a Cycle Period

Sensitivity in a piezoresistive pressure sensor refers to the degree to which the sensor’s electrical resistance changes in response to an applied pressure. Sensitivity quantifies how effectively the sensor can detect and respond to variations in external force. In this research, the sensitivity of the prepared piezoresistive sensors was evaluated through the half cycle period of the loading condition. The sensitivity (S) of the sensor material, in terms of piezo resistance, was determined using the equation for every sensor with respect to pressure, as outlined below:

$$S=\frac{△R/R_{o}}{△P}$$

where △*R* = change in resistance, *R*_0_ = resistance in initial state, and △*P* = change in applied pressure.

**Figure 11:**
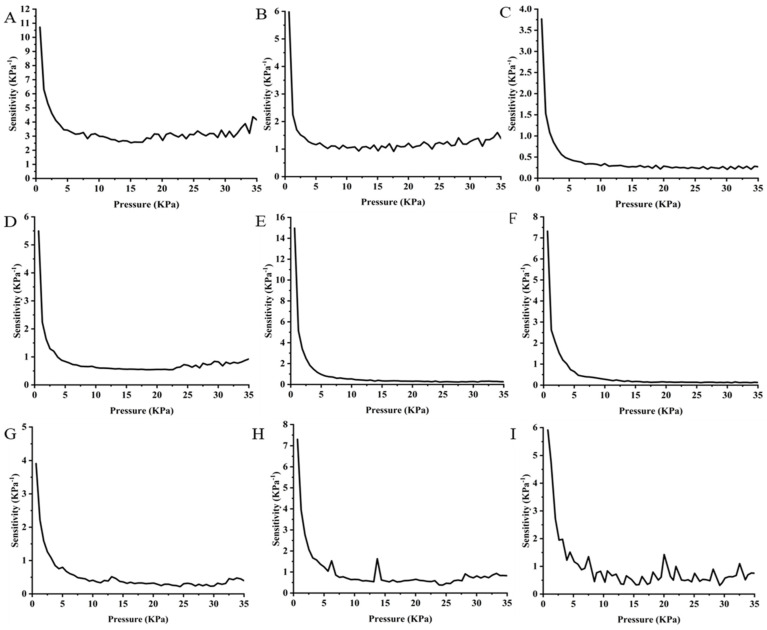
Sensitivity of the graphene-impregnated piezoresistive pressure sensor with respect to pressure variation over the half-cycle period: (**A**) the drop-casted sensing layer with the silver-coated knitted electrode, (**B**) the drop-casted sensing layer with the powder-bonded silver-coated nonwoven-electrode, (**C**) the drop-casted sensing layer with the spun-bonded silver-coated nonwoven electrode, (**D**) the electro-spun sensing layer with the silver-coated knitted electrode, (**E**) the electro-spun sensing layer with the powder-bonded silver-coated nonwoven electrode, (**F**) the electro-spun sensing layer with the spun-bonded silver-coated nonwoven electrode, (**G**) the electro-sprayed sensing layer with the silver-coated knitted electrode, (**H**) the electro-sprayed sensing layer with the powder-bonded silver-coated nonwoven electrode, and (**I**) the electro-sprayed sensing layer with the spun-bonded silver-coated nonwoven electrode.

The sensitivity of the graphene-impregnated piezoresistive pressure sensors fabricated with three different types of electrodes is shown in Figure 11. The sensitivity of the piezoresistive pressure sensor is seen to reduce as the pressure increases. On the fully compressed state, there is a reduced chance of creating additional electrical paths within the graphene-impregnated nonwoven material. Additionally, the conductive networks tend to be saturated, resulting in a marginal decrease in electrical resistance [21]. In the drop-casted sensing layers, the sensitivity in the sliver-coated spun-bonded electrode has smooth change, and the sensitivity value is gradually reduced due to the applied pressure. The maximum sensitivity in the drop-casted sensor fabricated with silver-coated knitted is 10.5 KPa^−1,^ and the minimum sensitivity is 2.5 KPa^−1^. The highest sensitivity value of the drop-casted sensors is considerably more optimum than other reported conductive materials (graphene, Mexene, silver)-based nonwoven sensors [11,12,14,15]. The maximum sensitivity results from the highest amount of graphene content as well as more frequent contact between neighbouring graphene and silver particles in the electrode. In the case of the electro-spun sensing layer, the silver-coated powder-bonded electrode exhibits the highest sensitivity among the tested electrodes. The nanoporous electro-spun structure can create more frequent contact with the powder-bonded electrode. In silver-coated spun-bonded electrodes, the sensitivity is lower than that of powder-bonded electrodes. In the sliver-coated knitted electrode, initial sensitivity is lower compared to the other two electrodes, but the minimum sensitivity value is 0.9 KPa^−1^ at higher pressure, and in the other two electrodes, powder bonded (0.2 KPa^−1^) and silver coated spun bonded (0.1 KPa^−1^). Though the sensitivity of the powder-bonded and spun-bonded electrode at lower pressure is comparatively higher by considering the overall performance on the consideration of the advantageous application, the silver-coated knitted electrode is suitable for both small and high-pressure applications, and the powder-bonded electrode is suitable in the low-pressure application. According to the value of sensitivity in the electro-sprayed sensing layer with the powder-bonded silver-coated electrode (7.3 KPa^−1^) is higher compared to the spun-bonded silver-coated electrode (5.8 KPa^−1^) and the silver-coated knitted electrode (3.8 KPa^−1^). The sensitivity of all the electrode materials showed the relations as follows: the drop-casted piezoresistive sensing layer with the silver-coated knitted electrode (10.5 KPa^−1^) > the electro-sprayed sensing layer fabricated with the powder-bonded silver-coated electrode (7.3 KPa^−1^) > the electro-spun sensing layer with the sliver-coated knitted electrode (5.5 KPa^−1^). The drop-cast sensing layer with the sliver-coated knitted electrode has superior performance among all the sensors due to their graphene content (0.146 g) in the sensing layer. The silver-coated knitted electrode provides superior sensing performance, stable signal quality, and reliable connectivity compared to the nonwoven alternatives as the knitted electrode can ensure a quick connection to produce a signal as the knitted structure has a strong dependence on generating a resistance signal upon elongation of the materials [19]. On the other hand, random and irregular fibre orientation on the powder-bonded and spun-bonded electrodes leads to irregular response results from irregular contact between silver and graphene particles during loading and unloading conditions [20]. It can be inferred that even a small pressure change in the silver-coated knitted electrode will result in a significant change in the output signal, and the lower sensitivity in the powder-bonded and spun-bonded silver-coated electrode indicates larger pressure needed to change in the output signals. As graphene density in the drop-casted sensing layer and the silver-coated knitted electrode have more flexibility for making connections with the sensing layer, more graphene density can facilitate frequent contact between particles within the small pressure and create changes in the output signal. From the sensing behaviour, the silver-coated knitted electrode performed the best compared to the other two electrodes.

## 4. Conclusions

In summary, this study successfully developed graphene-impregnated nonwoven-based piezoresistive pressure sensors and systematically evaluated their design, sensory behaviour, and electromechanical performance. Experimental findings revealed that all fabricated sensors exhibited resistance changes under compressive loading, attributed to the increased contact between graphene particles, facilitating the formation of conductive pathways. Among the tested sensors, drop-casted graphene-based sensing layers with silver-coated knitted electrodes exhibited superior resistance recovery, stable signal output, and minimal hysteresis during cyclic loading, highlighting their suitability for high-performance pressure sensing applications. Conversely, sensors fabricated via electrospinning and electro-spraying demonstrated higher resistance fluctuations and lower sensitivity, with a progressive decline in resistance recovery over repeated cycles. These variations were attributed to differences in graphene particle distribution, porosity of the sensing layer, and electrode–substrate interactions. This study emphasises the critical role of electrode selection and sensing layer preparation in optimising sensor response, stability, and durability. The porous nature of nonwoven surfaces significantly influenced graphene dispersion, while the mechanical flexibility of textile-based electrodes ensured sensor functionality after multiple deformation cycles. Furthermore, the research highlights the need for careful control of graphene content within the sensing layers to enhance piezoresistive performance and ensure consistent electromechanical response. Future work should focus on fine-tuning fabrication parameters, including applied voltage, ink flow rate, fibre diameter, nonwoven GSM, silver content, and layer thickness, to further enhance the sensitivity, stability, and robustness of graphene-based piezoresistive sensors. Additionally, in situ analysis of graphene particle movement under mechanical stress could provide a deeper understanding of the piezoresistive mechanism, aiding in the development of next-generation smart textile-based pressure sensors for wearable electronics, biomedical monitoring, and industrial applications.

## Figures and Tables

**Table 1:** Details of specifications of the textile-based silver-coated electrodes.

Electrode	Thickness (mm)	Areal Density (g/m^2^)	Electrical Resistance	Source
Knitted fabric	0.38	140	<100 Ω/Sq.	HITEK LTD
Spunbonded Nonwoven	0.1	60	<100 Ω/ Sq.	HITEK LTD
Powder-bonded Nonwoven	0.15	70	<100 Ω/ Sq.	HITEK LTD

**Table 2:** Electrospinning and electro-spraying parameters.

Solutions	Flow Rate (ml//hr.)	Voltage (KV)	Needle Gauge (Number of Needles/Inch)	Working Distance (cm)	Collector Speed
Polyurethane Diol	5	25	18	20	250
Graphene Ink	3	25	18	20	250

**Table 3:** Details specifications of the piezoresistive pressure sensors.

Piezoresistive Sensing Layer	Thickness (mm)	Areal Density (g/m^2^)	Dimension (Length × Width)	Graphene Content (g)	Fibre/Polymer
Drop casted	0.38	305	50 mm × 50 mm	0.146	Polycaprolactone
Electro-spun	0.60	196	50 mm × 50 mm	0.094	Polycaprolactone
Electro-sprayed	0.20	48	50 mm × 50 mm	0.022	Polypropylene /Polyester

**Table 4:** Analysis of variance (ANOVA) of the initial resistance of prepared sensors.

Initial Resistance	Source of Variation	SS	df	MS	F-Value	p-Value
Drop casted sensors	Between Groups	0.035	2	0.0178	7.778504	0.006
	Within Groups	0.027	12	0.002		
	Total	0.063	14			
Electro-spun sensors	Between Groups	3.835	2	1.917	16.328	0.001
	Within Groups	1.409	12	0.117		
	Total	5.244	14			
Electro-spraying sensors	Between Groups	33,403.6	2	16,701.8	749.519	0
	Within Groups	267.4	12	22.283		
	Total	33,671	14			

SS = Sum of Squares, df = degree of freedom, and MS = Mean Square.

## Data Availability

Data will be available on request.
